# Coding the issue: low anterior resection syndrome following rectal cancer treatment

**DOI:** 10.3389/fsurg.2024.1503410

**Published:** 2024-11-18

**Authors:** Cameron N. Fick, Samantha M. Linhares, Kurt S. Schultz, Andrew C. Esposito, Nathan A. Coppersmith, Haddon J. Pantel, Vikram B. Reddy, Ira L. Leeds, Anne K. Mongiu

**Affiliations:** Division of Colon and Rectal Surgery, Department of Surgery, Yale School of Medicine, New Haven, CT, United States

**Keywords:** rectal cancer, low anterior resection syndrome, bowel dysfunction, ICD-10 classification, quality of life

## Introduction

Over the past 20 years, significant advances in the surveillance and management of rectal cancer have been achieved (1, 2). Concerning surgical management, a sphincter-preserving low anterior resection with total mesorectal excision has become the standard of care whenever technically feasible (1). However, even the most technically sound resection can negatively affect patients following surgery, with 80%–90% of patients developing bowel dysfunction after surgery, known as low anterior resection syndrome (LARS) (3–5). LARS encompasses a constellation of symptoms ranging from fecal incontinence, clustering, urgency, or pain with defecation and ultimately significantly impacts the long-term quality of life (QoL) (6, 7). Symptoms related to LARS are most likely to improve within the first year postoperatively, but for those who do not have improvement, symptoms are likely become chronic (5, 8). As such, patients will require long-term follow-up, and may have to trial various treatment options to optimize their QoL.

In medicine, the universal language used to communicate diagnoses, symptoms, and procedures is the World Health Organization's International Classification of Diseases (ICD) system, now in its tenth version (ICD-10) (9). The United States version of this system was created by the Centers for Medicare and Medicaid Services (CMS) and National Center for Health Statistics in 2015 in the form of ICD-10-CM (Clinical Modification) for clinical diagnoses and ICD-10-PCS (Procedures Coding System) for procedures (10). The ICD-10 system is most frequently used for billing purposes, as the US healthcare system relies heavily upon these codes to justify treatment strategies to payers. This system is also a fundamental tool to track disease and evaluate healthcare outcomes; consequently, a disease process without an ICD-10 code is nearly impossible to identify accurately for quality improvement and research purposes.

There are 70,000 + billing codes in the ICD-10 system, representing common diseases treated by providers, such as hypertension (I10) and hyperlipidemia (E78.5) (9). There are also several unique billing codes represented, including sucked into the jet engine (V97.33XD), struck by a duck (W61.62XD), burned due to water skis on fire (V91.07XD). Instead of grouping these encounters under “general trauma”, having these detailed ICD-10 codes allows physicians and researchers to better understand the issues affecting distinct patient populations. Similarly, certain post-surgical morbidities are represented by discrete ICD-10 codes, including post-cholecystectomy syndrome (K91.5), which helps to characterize a specific subset of symptoms (9).

LARS is a well-documented side-effect of sphincter-sparing resection for rectal cancer with an unknown disease burden. Although LARS affects up to 80% of patients postoperatively, it does not have its own ICD-10 code (3–5, 11). The closest ICD-10 codes that currently serve as a surrogate for LARS are K91.89, “Other postprocedural complications and disorders of the digestive system,” or R19.8, “Other specified symptoms and signs involving the digestive system and abdomen” (9). Both codes are incredibly broad and could encompass numerous other diseases while missing the granularity needed to accurately capture LARS in administrative data. Without a unique ICD-10 code for LARS, proper evaluation and treatment are challenging. Given its prevalence and impact on patients, we aim to outline the importance of developing an ICD-10 code to promote better care for patients with LARS.

## Communication and reimbursement

Although LARS is a common condition, the awareness level of the true prevalence and most relevant aspects of patients’ QoL remains suboptimal. A survey study by Thomas et al. found that nearly 80% of colorectal surgeons and nurses believed less than 60% of patients would develop LARS postoperatively. Furthermore, less than half the surgeons used the LARS score to assess patients postoperatively (12). Patients in two different qualitative studies reflected that they felt they were not adequately prepared preoperatively for the likelihood of developing some component of permanent bowel dysfunction afterwards (13, 14). The ICD-10 system is a common language of communication for practitioners across various specialties as patients encounter different clinical settings. A study evaluating the online information available for LARS found that only 32% of appropriate websites defined LARS and only 40% listed all the symptoms under the scope of LARS (15). The opportunity to assign a patient with an ICD-10 code diagnosis could promote widespread awareness, allow different providers to more easily recognize this problem, and encourage efforts to characterize the disease more appropriately.

Once diagnosed with LARS, an associated ICD-10 code allows physicians to be more attentive during the work-up and care of their patients. Patient have reported they expect their surgeon and ancillary staff to initiate and prepare them adequately to manage these issues postoperatively (14). While the colorectal surgeon is reimbursed for the initial surgery and postoperative care, the complicated nature of LARS and the long-term follow-up required are not adequately reimbursed. Multiple treatment options including pelvic floor rehabilitation, transanal irrigation or sacral nerve stimulation have been described in previous studies but there is not one algorithm widely adopted as standard of care (16). The closest ICD-10 diagnoses for these treatment options could include diarrhea (R19.7) or incontinence of feces (R15.9) (9). However, using these misleading codes for LARS treatment modalities may delay or prevent patients from receiving the appropriate care due to insurance denial and prohibitive cost.

## Research and patient empowerment

To develop evidence-based interventions for LARS depends on having a large cohort of patients to analyze various risk factors, pathophysiology and outcomes. An ICD-10 code would provide researchers with a method to cluster patients across multiple health systems according to a diagnosis and allow easier identification of areas of possible intervention. Without an ICD-10 code, the true disease burden of LARS will remain unknown. This should be a key factor in securing research funding. A prior study found that National Institutes of Health funding, not disease burden, was the greatest predictor for future funding (17). Further advocacy should be done to appropriately allocate funds towards those diseases with a high disease burden and impact on QoL.

A large component of successful management of chronic disease depends on patient-driven engagement and self-care strategies (18). Providing patients with a label for their constellation of symptoms to connect them with other similar patients can help patients feel motivated to contribute to their own care. Patients with LARS have commented on wishing they had a support network with others who understand the same symptoms that they are going through (14). A pilot mixed methods study evaluating the feasibility and experience of an online patient-centered application for patients suffering from LARS found that this intervention helped address gaps in care and provide emotional support (19). Creating a diagnosis is an easy way to facilitate patients to develop support networks and provide each other with psychosocial support.

Furthermore, there is a social stigma around discussions of bowel habits, leading to many patients hesitating to bring up the topic with their providers. Patients have discussed they would attempt to solve problems on their own (14). Prior studies have shown that patients who received a diagnosis for symptoms related to multiple sclerosis had a positive patient reaction and increased satisfaction, and for those with confirmed diagnosed asthma were more likely to have better mediation adherence (20, 21). An ICD-10 code to provide patients with a definitive diagnosis for their constellation of symptoms may alleviate the stigma around discussing LARS and bowel habits, thus allowing a better rapport between providers and patients.

## Discussion

Creating an ICD-10 code for LARS would significantly impact the recognition, reimbursement, and research efforts surrounding this syndrome. In an era of patient-driven outcomes and QoL metrics, many patients suffering from LARS have been left behind. LARS can have a profound impact on QoL for years following surgical resection of rectal cancer (8, 22). The benefits of developing an ICD-10 are multifaceted, including optimizing patient care for easier reimbursement and treatment, facilitating more accessible data collection to investigate the pathophysiology and symptomatology of LARS, and empowering patients to speak about their symptoms and develop a more robust support network. This opinion paper hopes to galvanize the CMS to create an ICD-10 code for LARS to improve medical care for thousands of patients suffering from the treatment side effects of rectal cancer.
